# Altered TRPM7-Dependent Calcium Influx in Natural Killer Cells of Myalgic Encephalomyelitis/Chronic Fatigue Syndrome Patients

**DOI:** 10.3390/biom13071039

**Published:** 2023-06-26

**Authors:** Stanley Du Preez, Natalie Eaton-Fitch, Peter K. Smith, Sonya Marshall-Gradisnik

**Affiliations:** 1 National Centre for Neuroimmunology and Emerging Diseases, Menzies Health Institute, Griffith University, Gold Coast 4215, Australia; 2 Consortium Health International for Myalgic Encephalomyelitis, Menzies Health Institute Queensland, Griffith University, Gold Coast 4215, Australia; 3 School of Pharmacy and Medical Sciences, Griffith University, Gold Coast 4215, Australia; 4 School of Medicine and Dentistry, Griffith University, Gold Coast 4215, Australia; 5 Queensland Allergy Services, Gold Coast 4215, Australia

**Keywords:** myalgic encephalomyelitis, chronic fatigue syndrome, natural killer cell, transient receptor potential melastatin 7, calcium

## Abstract

Myalgic encephalomyelitis/chronic fatigue syndrome (ME/CFS) is a disabling multisystemic condition. The pathomechanism of ME/CFS remains unestablished; however, impaired natural killer (NK) cell cytotoxicity is a consistent feature of this condition. Calcium (Ca^2+^) is crucial for NK cell effector functions. Growing research recognises Ca^2+^ signalling dysregulation in ME/CFS patients and implicates transient receptor potential ion channel dysfunction. TRPM7 (melastatin) was recently considered in the pathoaetiology of ME/CFS as it participates in several Ca^2+^-dependent processes that are central to NK cell cytotoxicity which may be compromised in ME/CFS. TRPM7-dependent Ca^2+^ influx was assessed in NK cells isolated from n = 9 ME/CFS patients and n = 9 age- and sex-matched healthy controls (HCs) using live cell fluorescent imaging techniques. Slope (*p* < 0.05) was significantly reduced in ME/CFS patients compared with HCs following TRPM7 activation. Half-time of maximal response (*p* < 0.05) and amplitude (*p* < 0.001) were significantly reduced in the HCs compared with the ME/CFS patients following TRPM7 desensitisation. Findings from this investigation suggest that TRPM7-dependent Ca^2+^ influx is reduced with agonism and increased with antagonism in ME/CFS patients relative to the age- and sex-matched HCs. The outcomes reported here potentially reflect TRPM3 dysfunction identified in this condition suggesting that ME/CFS is a TRP ion channelopathy.

## 1. Introduction

Myalgic encephalomyelitis/chronic fatigue syndrome (ME/CFS) is a debilitating multisystemic condition that demonstrates post-exertional neuroimmune exhaustion as its cardinal feature [1,2]. Symptoms reflecting immune, autonomic, cardiovascular, gastrointestinal, endocrine, metabolic, and cognitive dysfunction also appear in ME/CFS [3,4,5]. The clinical profiles and the illness severity vary, with approximately 25–29% of patients being severely affected [6]. However, no standardised diagnostic test or accepted pharmacotherapeutic for ME/CFS exists as the pathomechanism of this condition remains undefined [7]. Patient diagnosis therefore currently relies on various clinical case definitions, including the International Case Criteria (ICC, 2011) [1] and the Canadian Consensus Criteria (CCC, 2003) [8]. Research has identified natural killer (NK) cells as a reliable cellular model to investigate ME/CFS as NK cell dysfunction is a consistent feature of this condition [9].

NK cells are innate lymphoid cells that contribute to immunosurveillance and the elimination of target cells that are infected with intracellular pathogens or displaying malignant changes [10,11]. Recognition of target cells and subsequent elimination is determined by an overall activating signal communicated downstream of immunoreceptor tyrosine-based activation motif phosphorylation [12]. Once a target cell is identified, an immune synapse forms with the target. Cytoskeletal rearrangements within the NK cell direct the polarisation of cytotoxic granules toward the immune synapse [13]. Perforin and granzyme are released from secretory lysosomes and subsequently catalyse the death of the target cell [14]. The precise regulation of intracellular calcium (Ca^2+^) is essential to enable these events [15].

Ca^2+^ acts as a messenger molecule in the signalling cascades of numerous biological processes and is required for all cell types to function [16,17]. Transcription, proliferation, differentiation, metabolism, apoptosis, and effector functions demand Ca^2+^ at specific concentrations to occur correctly [18]. Hence, several mechanisms ensure that intracellular Ca^2+^ concentrations and homeostasis are well controlled. Previous investigations have identified Ca^2+^ dysregulation and impaired cytotoxic activity in the NK cells of ME/CFS patients [19,20,21,22,23,24,25]. Research has indicated that the dysfunction of members belonging to the transient receptor potential (TRP) superfamily of Ca^2+^-permeable ion channels may be responsible for these observations.

TRPs are polymodal sensors that detect environmental stimuli and mediate adaptive cellular responses through the influx of cations [26]. All tissues express TRPs, with each cell type bearing a unique signature according to its physiological role and environmental conditions [27]. Six subfamilies are described in mammals, with TRP melastatin (TRPM) members garnering particular interest in ME/CFS research. Using the NK cells of ME/CFS patients, numerous single nucleotide polymorphisms were identified across TRP ion channels including sub-members for the canonical (C), melastatin, and vanilloid channel families [28].

Subsequent flow cytometric, patch-clamp electrophysiology, and microscopy investigations revealed a loss of TRPM3 ion channel function, reduced surface expression, and impaired Ca^2+^ mobilisation in the NK cells of ME/CFS patients compared with healthy controls (HCs) [19,20,21,23,24,25]. Interestingly, the expression levels of a related protein, TRPM2, were elevated on the NK cell membranes of ME/CFS patients according to flow cytometric analyses [22]. Therefore, TRPM3 dysfunction and associated Ca^2+^ dysregulation may manifest as changes to other TRPM channels in the form of compensatory mechanisms.

The Ca^2+^- and magnesium (Mg^2+^)-permeable channel-kinase, TRPM7, was examined in a previous study to extend this hypothesis. Pharmacological inhibition of TRPM7 resulted in significantly higher co-localisation with cortical actin than pharmacological channel activation in ME/CFS patients [29]. This was not observed for HCs. Additionally, the co-localisation of TRPM7 with phosphatidylcholine-4,5-bisphosphate (PIP_2_) following antagonism treatment appeared to be greater in healthy participants than in those with ME/CFS [29]. These results suggest that TRPM7 may be altered in ME/CFS and thus implicate TRPM7 in the NK cell dysfunction and pathomechanism of this condition. However, it remains unclear from this initial investigation whether TRPM7 itself is functionally impaired, or if the results reflect known TRPM3 dysfunction and Ca^2+^ dysregulation in the NK cells of ME/CFS patients.

The present study was designed to evaluate TRPM7-dependent Ca^2+^ influx and elucidate whether the function of the ion channel component of this protein is altered in ME/CFS compared with HCs. This is the first investigation to examine the speed and maximum response of Ca^2+^ influx mediated via TRPM7 in the NK cells of ME/CFS patients and HCs. Fluorescence live-cell imaging techniques were used to measure these parameters. Outcomes generated from this study will inform future directions to characterise TRPM7 ion channel function and kinase activity. Furthermore, this study contributes to understanding the pathomechanism of ME/CFS and the identification of potential therapeutic targets to assist people affected by this condition.

## 2. Materials and Methods

### 2.1. Study Participants

ME/CFS patients and HCs were recruited through the National Centre for Neuroimmunology and Emerging Diseases (NCNED) patient database for this investigation. A comprehensive online questionnaire was administered to screen and define ME/CFS patients according to the ICC [1] and CCC [8] case definitions. ME/CFS patients that fulfilled the ICC or CCC case definitions and reported receiving a diagnosis of ME/CFS by a qualified physician were deemed eligible for this project. Nine ME/CFS patients from south-east Queensland and northern New South Wales were included in this study. Each ME/CFS patient was age- and sex-matched with a HC. The HC cohort was defined as non-fatigued individuals without any diagnosed illnesses. All participants were between 18 and 65 years of age, had a body mass index (BMI) between 18.5 and 30 kg/m^2^, and were non-smokers.

Exclusionary criteria were applied to all potential participants and included chronic illness (including autoimmune, cardiac, endocrine, metabolic, or primary psychiatric disorders), malignancy, alcohol misuse, pregnancy, breastfeeding, or administering pharmacological agents that are known to directly or indirectly affect TRPM7 channel-kinase activity, Ca^2+^ signalling, or immune cell activity. All participants provided written informed consent to partake in the present study. Ethics approval was obtained from the Gold Coast Human Research Ethics Committee (HREC/2019/QGC/56469) and the Griffith University Human Research Ethics Committee (GU/2019/1005).

Eligible participants donated 84 mL of whole venous blood, which were collected in ethylenediaminetetraacetic acid (EDTA) tubes between 7:00 a.m. and 11:00 a.m. in the south-east Queensland and Northern New South Wales regions of Australia. Each sample was de-identified by an independent researcher using a unique alpha-numeric code. A full blood count (FBC) was performed within 4 h of collection for each participant as a routine measure for exclusionary haematological abnormalities, including features of anaemia or white cell derangements.

All participants completed an online questionnaire that collected sociodemographic data, medical history, medications, and symptom history of the ME/CFS patients. ME/CFS symptom survey responses were grouped into 10 symptom categories including: (i) cognitive difficulties (slowed thought, impaired concentration, and memory consolidation issues); (ii) pain (headaches, muscle pain, and multi-joint pain); (iii) sleep disturbances (reversed sleep cycle, disturbed sleep cycle, and unrefreshing sleep); (iv) neurosensory disturbances (sensitivity to touch, vibration, taste, odour, sound; and poor coordination or balance); (v) immune disturbances (flu-like symptoms, sore throat, and tender lymph nodes); (vi) gastrointestinal disturbances (abdominal pain, nausea, and bloating); (vii) cardiovascular disturbances (orthostatic intolerances, light-headedness, and heart palpitations); (viii) respiratory disturbances (difficulty breathing, and air hunger); (ix) thermostatic instability (abnormal sweating episodes, hot flushes, and cold extremities); and (x) urinary disturbances (changed urination frequency and painful bladder). In addition, a 36-item short-form health survey (SF-36) and the World Health Organization (WHO) Disability Assessment Schedule (DAS) were used to determine the level of disability and quality of life (QoL).

### 2.2. Peripheral Blood Mononuclear Cell and Natural Killer Cell Isolation

Eighty mL of whole blood was used for peripheral blood mononuclear cells (PBMC) isolation via density gradient centrifugation using Ficoll (GE Healthcare, Uppsala, Sweden) as previously described [28]. PBMCs were stained with trypan blue (Invitrogen, Carlsbad, CA, USA) to determine cell count and cell viability. PBMCs were adjusted to a final concentration of 5 × 10^7^ cells/mL for NK cell isolation.

NK cells were isolated via immunomagnetic selection using the EasySep Negative Human NK cell Enrichment Kit (Stem Cell Technologies, Vancouver, BC, Canada). A fraction of the NK cell isolate was used to assess purity using flow cytometry. The NK cell population was defined by CD3^−^CD56^+^ surface expression. NK cells (1 × 10^5^) were labelled with CD3 PE-Cy7 (5 µL/test) and CD56 APC (20 µL/test) monoclonal antibodies (Becton Dickinson [BD] Biosciences, San Jose, CA, USA) for 20 min at room temperature (RT). Cells were then acquired at 10,000 events using the Accuri C6 flow cytometer (BD Biosciences, San Diego, CA, USA). The average NK cell purity for ME/CFS patients was 93.4 ± 0.7 and for HCs was 94.3 ± 1.0 in this study.

### 2.3. Calcium Imaging

All suspensions were prepared in, and washed with 1.8 mM Ca^2+^ buffer, unless otherwise stated. The Ca^2+^ buffer was prepared in milliQ water and contained NaCl 140 mM, KCl 5.4 mM, CaCl_2_ 1.8 mM, MgCl_2_ 1.0 mM, and HEPES 10 mM (Thermofisher, Waltham, MA, USA). The pH was adjusted to 7.40 ± 0.05 using NaOH and the osmolality was adjusted to 300 ± 10 mOsm/L using D-glucose (Thermofisher, Waltham, MA, USA).

NK cells were adhered to 24-well µ-plates (Ibidi, Lochhamer Schlag, Germany) using Corning ^®^ Cell-Tak TM Cell and Tissue Adhesive (BD Biosciences, San Jose, CA, USA) as previously described [24]. Next, NK cells were incubated using 1 µM Fluo-8-acetoxymethyl ester (AM) (Abcam, Cambridge, UK) and 0.02% Pluronic F127 (Thermofisher, Waltham, MA, USA) for 30 min at 37 °C. Cells were then washed and incubated for a further 20 min at RT to allow the de-esterification of Fuo-8-AM.

Fluo-8-loaded cells were imaged using the Nikon A1R microscope (40× objective) and fluorescence emissions were recorded using the iXon Life 888 Electron Multiplying CCD camera. The effects of naltriben (NTB) (Sigma-Aldrich, St. Louis, MO, USA), a TRPM7 ion channel agonist, and NS8593 (Sigma-Aldrich, St. Louis, MO, USA), TRPM7 channel-kinase antagonist, on TRPM7-dependent Ca^2+^ influx were examined using two separate protocols. The use of NTB and NS8593 is supported by previous research [30,31,32,33,34,35,36,37,38]. NTB modulates TRPM7 channel gating and promotes TRPM7-mediated Ca^2+^ entry with limited off-target effects [32]. Recently, the binding site of NTB was identified at the inter-subunit interface of TRPM7 tetramers formed by the fourth melastatin homology region of the TRPM7 protein [39]. The same investigation identified the binding site of NS8593 involving the N-terminal portion of S3, C-terminal portion of S4, S4-S5 linker, and the TRPM helix, which locks TRPM7 in its closed conformation [39]. Appropriate concentrations of NTB and NS8593 for this investigation were determined using dose response analysis (Supplementary Figures S1 and S2). All drugs were prepared as suspensions and administered via continuous perfusion methods. Acquisition was performed from the centre of individual wells adjacent to the perfusing line with the aspirating line positioned opposite. Recordings were obtained after flow-induced Ca^2+^ influx had stabilised. Firstly, baseline Ca^2+^ was obtained for 2 min before cells were stimulated with 40 µM NTB for 3 min, followed by 1 µM ionomycin for 4 min. In a separate well, fluorescence was recorded for 3 min in the presence of 6 µM NS8593 to desensitise TRPM7, followed by 6 µM NS8593 + 40 µM NTB for 3 min, then 40 µM NTB alone for 3 min, and finally 1 µM ionomycin for 4 min.

Fluorescence recordings were evaluated using NIS Research Elements (Nikon, NIS-Elements V5.2, Tokyo, Japan). Three measures of Ca^2+^ influx were determined. Amplitude represents the difference in the minimum and the maximum values of TRPM7-dependent Ca^2+^ influx corresponding to NTB stimulation. From this curve, the half-time of maximum response (T1/2) was also determined. The magnitude of TRPM7-dependent Ca^2+^ influx was calculated from the initial slope of the curve using OriginLabs. The initial slope is defined in this investigation as the first positive deflection within the first 15 s of the response curve that correlates with the application of NTB or ionomycin and reflects the rate of entry for Ca^2+^ influx [40]. All measurements were normalised against the ionomycin response curves to ascertain the proportion of the baseline, NTB, and NS8593 responses for individual cells.

### 2.4. Statistical Analysis

Statistical analysis was conducted using GraphPad Prism V8 (Graphpad Software Inc., Version 8, La Jolla, CA, USA) and OriginLabs (OriginLab Corporation, Northampton, MA, USA). Significance was set at *p* < 0.05. Normality was assessed using the Shapiro–Wilk test. Definitive outliers were identified using the robust regression and outlier removal method available in GraphPad Prism V8 and were defined as data points that were not represented within the distribution with a false discovery rate set at 0.1%. Cell recordings that returned an outlier value for a particular parameter had all its corresponding values excluded for slope, T1/2, and amplitude to minimise variability. Statistical differences were tested using the Mann–Whitney U non-parametric T test. All data are presented as the mean ± standard deviation (SD) unless otherwise stated.

## 3. Results

### 3.1. Participant Characteristics

A total of nine ME/CFS patients and nine age- and sex-matched HCs participated in this study between December 2021 and July 2022. All ME/CFS patients satisfied the CCC case definition and had no other conditions that would explain the symptoms. The participants’ demographic data are presented in Table 1. The average age for the ME/CFS cohort was 40.3 ± 4.5 years and it was 39.8 ± 4.0 years for the HC cohort. All participants were female. The average BMI of the ME/CFS cohort was 23.7 ± 1.1 kg/m^2^ and 22.0 ± 0.7 kg/m^2^ for the HC cohort.

QoL was assessed for all participants using the SF-36 and WHODAS inventories and presented in Table 1. SF-36 scores approaching 100 reflect good QoL, whereas scores towards 0 indicate poor QoL for the domain measured. Conversely, WHODAS scores that are closer to 0 suggest no limitations in functioning and values approaching 100 reflect severe functional impairments or disability for the corresponding domain. Mean WHODAS scores collected for the present investigation globally reflect greater impairments in the ME/CFS cohort compared with HCs and were statistically significant in all domains. Similarly, SF-36 inventory comparisons between groups demonstrate significantly reduced scores in the ME/CFS cohort compared with the HCs, excluding the wellbeing measure. The FBC parameters of both groups were within normal range and not significantly different.

### 3.2. Effect of Naltriben Treatment on Calcium Influx

**Figure 1:**
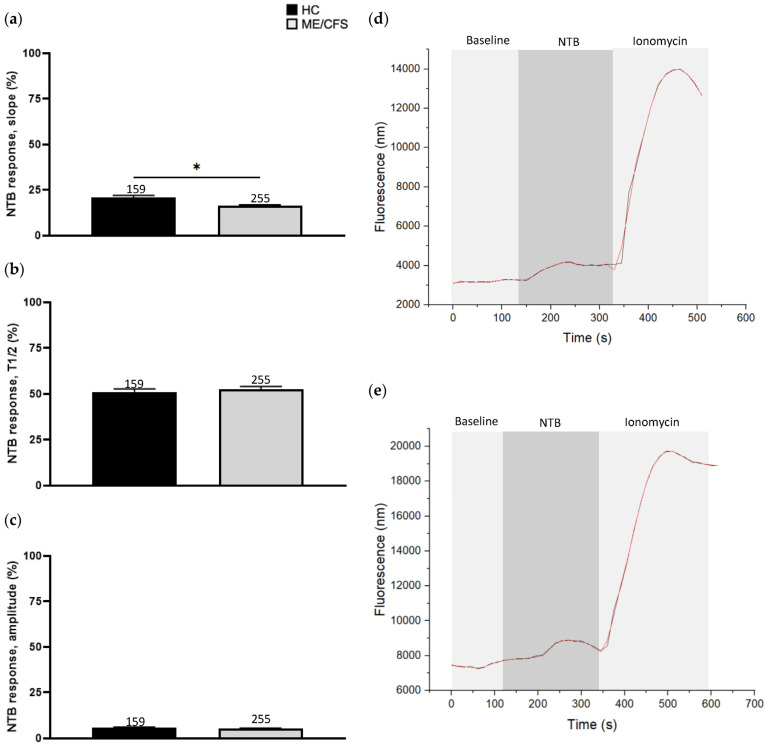
Ca^2+^ influx parameters from the stimulation of NK cells using 40 µM NTB. (**a**) Comparison of pooled slope values. (**b**) Comparison of pooled T1/2 values. (**c**) Comparison of pooled amplitude values. (**d**) Example Ca^2+^ influx recording with 40 µM NTB for TRPM7 activation and 1 µM ionomycin for non-specific Ca^2+^ influx in a HC. (**e**) Example Ca^2+^ influx recording with 40 µM NTB for TRPM7 activation and 1 µM ionomycin for non-specific Ca^2+^ influx in a ME/CFS patient. Data were collected from n = 9 HCs matched to n = 9 ME/CFS patients. The Ca^2+^ influx parameters were calculated from the smoothed (red) graph, which was generated by smoothing the raw (black) recording in OriginLabs displayed in (**d**,**e**). Values obtained from NTB stimulation for each cell were normalised to the ionomycin response curve within the same recording. The total number of cells analysed following the exclusion of definitive outliers (number of recordings excluded: HC = 14, ME/CFS = 25) are presented above the corresponding bar graph. All data are presented as mean ± SD with significance as determined using independent Mann–Whitney U tests. * *p* < 0.05. Abbreviations: calcium (Ca^2+^), healthy control (HC), myalgic encephalomyelitis/chronic fatigue syndrome (ME/CFS), natural killer (NK), naltriben (NTB), half-time of maximum response (T1/2).

NTB (40 µM) was used to stimulate TRPM7-mediated Ca^2+^ influx of isolated human NK cells (Figure 1). Amplitude (HC 6.0 ± 0.3, ME/CFS 5.4 ± 0.2) and T1/2 (HC 51.1 ± 1.7, ME/CFS 52.7 ± 1.4) were non-significant between the two groups. Slope recordings were lower in ME/CFS (16.2 ± 0.7) patients compared with the HCs (20.9 ± 1.2) and this difference was statistically significant (*p* < 0.05) (Figure 1).

### 3.3. Effect of NS8593 Treatment on Calcium Influx

**Figure 2:**
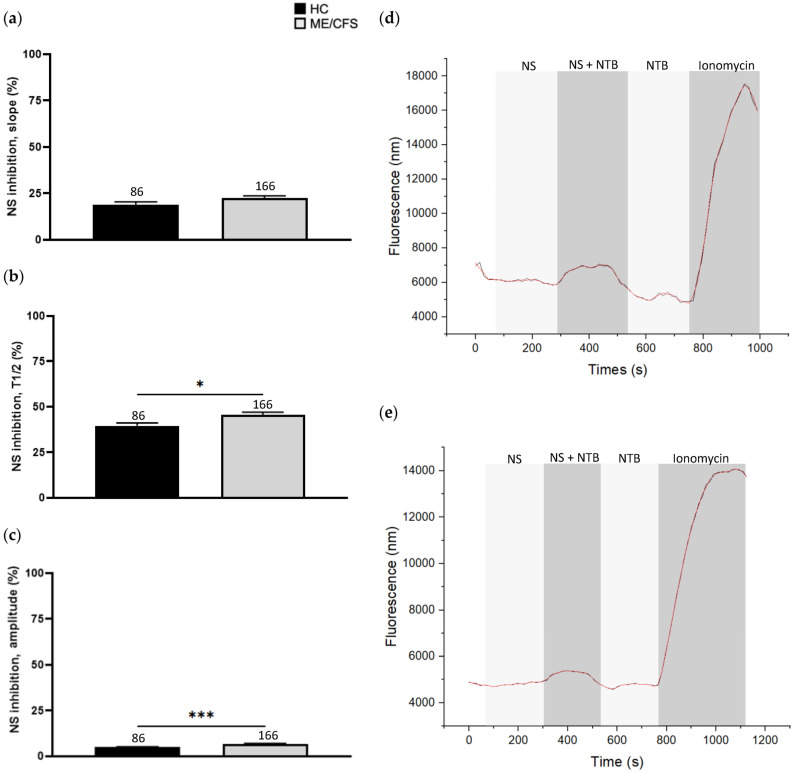
Ca^2+^ influx parameters from the inhibition and desensitisation of NK cells using 6 µM NS8593. (**a**) Comparison of pooled slope values. (**b**) Comparison of pooled T1/2 values. (**c**) Comparison of pooled amplitude values. (**d**) Example Ca^2+^ influx recording with 6 µM NS8593 for TRPM7 activation, 6 µM NS8593 + 40 µM NTB for TRPM7 desensitisation, followed by 40 µM NTB for TRPM7 activation and 1 µM ionomycin for non-specific Ca^2+^ influx in a HC. (**e**) Example Ca^2+^ influx recording with 6 µM NS8593 for TRPM7 activation, 6 µM NS8593 + 40 µM NTB for TRPM7 desensitisation, followed by 40 µM NTB for TRPM7 activation and 1 µM ionomycin for non-specific Ca^2+^ influx in a ME/CFS patient. Data were collected from n = 9 HCs matched to n = 9 ME/CFS patients. The Ca^2+^ influx parameters were calculated from the smoothed (red) graph, which was generated by smoothing the raw (black) recording in OriginLabs displayed in (**d**,**e**). Values obtained from NS8593 inhibition for each cell were normalised to the ionomycin response curve within the same recording. The total number of cells analysed following the exclusion of definitive outliers (number of recordings excluded: HC = 6, ME/CFS = 7) are presented above the corresponding bar graph. All data are presented as mean ± SD with significance as determined using independent Mann–Whitney U tests. * *p* < 0.05, *** *p* < 0.001. Abbreviations: calcium (Ca^2+^), healthy control (HC), myalgic encephalomyelitis/chronic fatigue syndrome (ME/CFS), natural killer (NK), NS8593 (NS), naltriben (NTB), half-time of maximum response (T1/2).

Inhibition of TRPM7 using NS8593 (6 µM) did not produce a significant difference in the slope of response between the ME/CFS (22.48 ± 1.22) and HC (19.05 ± 1.36) cohorts. Amplitude (4.97 ± 0.27, ME/CFS 6.68 ± 0.30) (*p* < 0.001) and T1/2 (HC 39.72 ± 1.61, ME/CFS 45.70 ± 1.52) (*p* < 0.05), however, were significantly different between the two groups and the HC cohort demonstrated a greater reduction in response relative to the recordings obtained from the ME/CFS patients (Figure 2).

## 4. Discussion

This pilot investigation is the first report on TRPM7-mediated Ca^2+^ influx in the NK cells of ME/CFS patients compared with HCs using a live cell Ca^2+^ imaging technique. Novel findings generated from this study include the demonstration of significantly reduced slope values in ME/CFS patients associated with the TRPM7 chemical agonist naltriben. Significantly impaired desensitisation of the channel-kinase from the T1/2 and amplitude values were also observed in the ME/CFS cohort.

The present investigation identified a significant reduction in slope values in the ME/CFS cohort following NTB stimulation. This parameter likely correlates with initial ion channel opening and stability [41], suggesting that the conformational change which opens and stabilises the TRPM7 ion channel pore may be delayed or impaired in ME/CFS. A previous investigation suggests that TRPC5 ion channel instability may impair Ca^2+^ influx and thus prevent the activation of Ca^2+^-dependent processes in neurons [42]. Sustained transmembrane Ca^2+^ influx is required to promote the translocation of secretory lysosomes, mitochondria, and the microtubule organising centre towards the immune synapse for NK cell degranulation to occur. Hence, TRPM7 instability may similarly constrain the sustained entry of Ca^2+^ through the ion channel domain, potentially impeding downstream events that are required for NK cell cytotoxic effector functions in ME/CFS patients. T1/2 and amplitude, however, were comparable between the two groups, indicating that peak Ca^2+^ influx mediated through TRPM7 and the time to achieve this is equivalent in ME/CFS patients and HCs.

By contrast, NS8593 treatment in the ME/CFS cohort indicated significantly reduced desensitisation of the TRPM7 channel-kinase according to the T1/2 and amplitude parameters relative to the HCs. Pooled slope values were statistically equivalent between the ME/CFS and HC cohorts. These findings indicate that the TRPM7 of ME/CFS patients is structurally or functionally resistant to desensitisation and may reflect a compensatory response to dysregulated cellular signalling or metabolic processes in ME/CFS.

As the TRPM7 stimulation and desensitisation protocols were structured differently, direct statistical comparison between the effects of NTB and NS8593 for each group could not be performed for this investigation. However, it is postulated that the findings of significantly greater slope values and significantly reduced T1/2 and amplitude values in the HC cohort compared with the ME/CFS patients collectively reflect underlying differences in the structure or function of TRPM7 in ME/CFS. Notably, pathophysiological changes to the microdomains where NTB or NS8593 act on TRPM7 may cause a reduced binding affinity for these drugs, or conformational instability of the TRPM7 ion channel may limit the duration of action of NTB and NS8593 in the NK cells of ME/CFS patients compared to HCs. Possible cellular and biochemical mechanisms that engage TRPM7 and possibly contribute to these observations in ME/CFS patients may also include impaired store-operated Ca^2+^ entry (SOCE), dysfunctional ATP production or utilisation in phosphorylation reactions, and abnormal interactions with PIP_2_ [29,43].

The present investigation employed the immunofluorescent live imaging technique to determine the changes in cytosol Ca^2+^ and there are certain limitations to it. Though it is important to recognise the limitations of this approach, appropriate pharmacological agents were used to address the concern of changes in Ca^2+^ independent of the TRPM7 ion channel activity. Methodological steps to mitigate this limitation include the use of the specific pharmacological agents NTB and NS8593 to activate and inhibit TRPM7-mediated Ca^2+^ influx, respectively. Furthermore, the EC50 for NTB of 40 µM and IC50 for NS8593 of 6 µM were determined using the protocol performed in the present study. This was to ensure that appropriate concentrations of each drug were used to limit potential off-target affects that may influence the results of the current investigation. Patch-clamp electrophysiology and ratiometric Ca^2+^ imaging is suggested as an additional complementary technique to validate our present findings and further characterise the potential changes in TRPM7-dependent Ca^2+^ influx in ME/CFS patients relative to HCs, using NTB and NS8593.

Results generated from this investigation support the findings of a previous study that reported significantly increased co-localisation of TRPM7 with cortical actin following desensitisation of the channel-kinase in ME/CFS patients compared with HCs [29]. Treatment of NK cells with NS8593 resulted in this difference, suggesting dysregulation involving both the TRPM7 ion channel and kinase components in ME/CFS rather than the ion channel moiety in isolation as the TRPM7 kinase domain regulates TRPM7 ion channel opening, actin dynamics, and translocation of the TRPM7 protein [43]. Alternatively, the observed differences in co-localisation may also be explained by structural or functional alterations to the microdomains that contribute to TRPM7 ion channel gating in ME/CFS patients where NTB and NS8593 are unable to modulate TRPM7 channel gating to the same degree compared with HCs.

Furthermore, desensitisation of TRPM7 with NS8593 significantly increased the co-localisation of this protein with PIP_2_ in both the ME/CFS and HC cohorts. Importantly, PIP_2_ is an essential cofactor for TRPM7 function [44] and the observations collected from this initial study were suggestive of a compensatory pathway to enhance TRPM7 activity in NK cells derived from ME/CFS patients and HCs. It was unclear whether this observation was due to a functional variation in the TRPM7 protein, or was a product of dysregulation of the Ca^2+^-permeable ion channel, TRPM3, observed in ME/CFS [29]. Accumulating evidence has identified TRPM3 dysregulation as a potential pathomechanism or a notable pathological feature of ME/CFS. Therefore, the findings of the present study of altered Ca^2+^ influx mediated by the TRPM7 channel-kinase may be additional to the TRPM3 anomalies observed in this condition.

Previous studies have identified significant genetic, phenotypic, and functional dysregulation of TRPM3 in the NK cells of ME/CFS patients relative to matched HCs [19,20,21,23,24,25,28,45]. Findings include significantly reduced surface expression and impaired Ca^2+^ influx assessed via patch-clamp electrophysiology, and, more recently, live cell imaging techniques. Both TRPM3 and TRPM7 have considerable structural and functional homology including the regulation of Ca^2+^- and kinase-signalling cascades [41,46].

Altered membrane Ca^2+^ influx can significantly impair cellular function and physiological processes [16,17,18,30]. Ca^2+^ is an essential cofactor for several NK cell activities including cytotoxicity [15,30,47,48,49,50]. Ligation of activating receptors at the NK cell surface promotes Ca^2+^ influx and Ca^2+^ store release to prolong the signalling cascade [51,52,53,54,55,56]. Moreover, the activation of various kinase cascade mediators including Ras, P38, phosphatidylinositol 4,5-bisphosphate 3-kinase, and mitogen-activated protein kinases is facilitated by Ca^2+^ [57,58]. Successful transduction of this cascade and associated pathways is required for NK cell cytotoxicity and cytokine production [59,60,61,62]. ME/CFS patients have previously demonstrated marked reductions in the phosphorylation of kinase signalling mediators in activated NK cells [63], indicating that changes in intracellular Ca^2+^ concentrations significantly alter the signalling capacity of these mediators in ME/CFS. Notably, PIP_2_ and TRPM7 are crucial molecules required for phosphorylation reactions [43].

TRPM7 participates in several additional cellular processes including the regulation of SOCE, Mg^2+^ homeostasis, and cellular energy homeostasis [43,64]. In non-excitable cells, including NK cells, SOCE is a major mechanism supplying Ca^2+^ for signalling cascades [65]. It is currently unclear whether SOCE is also compromised in ME/CFS patients. Further investigations to characterise SOCE and correlations with TRP channel dysfunction in the NK cells of ME/CFS patients is indicated as the SOCE signalling network may also be involved in the pathomechanism of ME/CFS. Assessment of the TRPM7 kinase would support these future investigations as the kinase domain is the principal moiety of TRPM7 that regulates SOCE. Quantification of TRPM7 surface expression is of interest to determine whether there is a relative abundance of TRPM7 to compensate for impaired TRPM7 ion channel stability, or secondary to the Ca^2+^ signalling dysregulation present in the NK cells of ME/CFS patients compared to HCs as reported for TRPM2 [22]. Conversely, a relative deficit of TRPM7 may exist due to compromised protein trafficking mechanisms and dysregulated Ca^2+^ signalling in the NK cells of ME/CFS patients compared with HCs as reports of impaired TRPM3 [20]. Furthermore, electrophysiological investigations of TRPM7 in the NK cells of ME/CFS patients would expand on the findings of the present study suggesting impaired TRPM7 channel-kinase function alongside TRPM3 dysfunction as an ion channelopathy in ME/CFS.

Clinical evidence further supports TRPM7 as a potential therapeutic target to improve health outcomes for ME/CFS patients. Aripiprazole is a promising therapeutic agent for the treatment of ME/CFS that is suggested to reduce microglial activation and associated neuroinflammation via TRPM7 [66]. A retrospective study of 101 ME/CFS patients observed a that 74% of the participants experienced a significant improvement in ME/CFS symptoms [67]. Validation of these findings with biological assays and clinical trials may corroborate the results of the present investigation proposing TRPM7 as an additional molecular mechanism and therapeutic target with TRPM3 in ME/CFS.

## 5. Conclusions

ME/CFS is a complex and disabling neuroimmune disorder with an emerging pathomechanism hypothesis implicating TRP ion channels. Several studies have demonstrated TRPM3 dysfunction in the NK cells of ME/CFS patients, which may alter the activity of other molecules, including TRPM7. The present study identified that Ca^2+^ influx mediated by TRPM7 is altered in ME/CFS patients relative to HCs. Further research to define the role of the TRPM7 channel-kinase in ME/CFS is therefore required to clarify the pathomechanism of this condition for diagnostic and therapeutic purposes.

## Figures and Tables

**Table 1:** Participants’ demographics, quality of life, disability scores, and full blood count results. Significance denoted by * (*p* < 0.05), ** (*p* < 0.01) and *** (*p* < 0.001).

Category	Item	HC	ME/CFS	*p*-Value
General demographics	Age (years)	39.8 ± 4.0	40.3 ± 4.5	0.7263
	Gender			
	Male (%, n)	0, 0	0, 0	
	Female (%, n)	100, 9	100, 9	
	BMI (kg/m^2^)	22.0 ± 0.7	23.7 ± 1.1	0.3789
WHODAS	Understanding and communication	8.3 ± 2.5	59.7 ± 8.4	***
	Mobility	2.8 ± 2.8	61.1 ± 7.3	***
	Self-care	0.0 ± 0.0	36.1 ± 9.3	**
	Interpersonal relationships	8.3 ± 3.6	47.2 ± 13.0	*
	Life activities	7.6 ± 3.7	73.6 ± 7.0	***
	Participation in work/school	4.9 ± 2.5	73.4 ± 10.3	***
	Participation in society	2.1 ± 0.7	64.2 ± 8.4	***
Illness demographic (SF-36)	Pain (%)	90.5 ± 3.8	50 ± 9.5	**
	Physical functioning (%)	98.9 ± 1.1	27.8 ± 7.7	***
	Role physical (%)	98.6 ± 1.4	15.3 ± 5.2	***
	General health (%)	72.9 ± 4.5	31.9 ± 5.2	*
	Social functioning (%)	94.4 ± 3.0	22.2 ± 8.0	***
	Role emotional (%)	92.6 ± 3.2	56.5 ± 12.3	*
	Wellbeing (%)	71.1 ± 3.2	57.2 ± 7.9	0.1868
	Vitality (%)	66.7 ± 3.9	6.9 ± 3.2	***
Full blood count	White blood cells	5.9 ± 0.4	6.4 ± 0.5	0.5353
	Lymphocytes	1.68 ± 0.12	1.92 ± 0.15	0.2005
	Neutrophils	3.59 ± 0.24	3.84 ± 0.41	0.5353
	Monocytes	0.46 ± 0.03	0.46 ± 0.03	0.8259
	Eosinophils	0.19 ± 0.04	0.14 ± 0.02	0.5687
	Basophils	0.03 ± 0.01	0.04 ± 0.01	0.5093
	Platelets	266 ± 13	250 ± 13	0.5961
	Red blood cells	4.34 ± 0.09	4.44 ± 0.10	0.7263
	Haematocrit	0.39 ± 0.01	0.40 ± 0.01	0.4009
	Haemoglobin	131 ± 3	135 ± 2	0.7949

## Data Availability

The data presented in this study are available on request from the corresponding author. The data are not publicly available due to privacy restrictions.

## Supplementary Materials

The following supporting information can be downloaded at: https://www.mdpi.com/article/10.3390/biom13071039/s1, Figure S1: Naltriben dose responses and EC50 determination; Figure S2: NS8593 dose responses and IC50 determination.
