# Supplementary material for: Altered TRPM7-Dependent Calcium Influx in Natural Killer Cells of Myalgic Encephalomyelitis/Chronic Fatigue Syndrome Patients
Source: Biomolecules. 2023 Jun 26;13(7):1039. doi: 10.3390/biom13071039 (PMC10377690; doi:10.3390/biom13071039)
Supplement: Supplementary file 1 [file biomolecules-13-01039-s001.zip › biomolecules-2367429-supplementary.pdf]

Parameter	Dose response curve	Calculated EC50
Slope	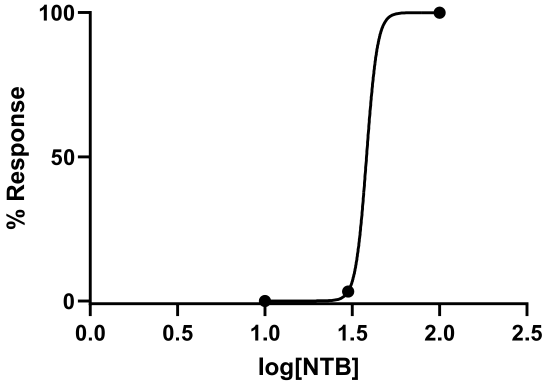	34.47 µM
T1/2	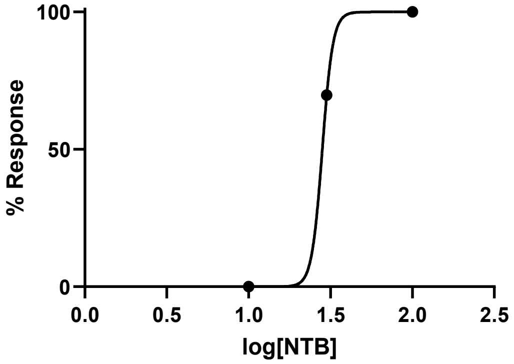	28.20 µM
Amplitude	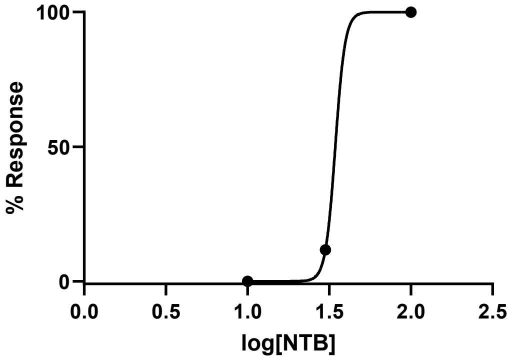	34.47 µM
	Average = 32.38 µM, final concentration used = 40 µM	

Figure S1. Naltriben dose responses and EC50 determination. Curves were produced using n = 4 HC not included in the main study. Abbreviations: half maximal effective concentration (EC50), naltriben (NTB), half-time of maximal response (T1/2).

<table>
<tr><th>Parameter</th><th>Dose response curve</th><th>Calculated IC50</th></tr>
<tr><td>Slope</td><td>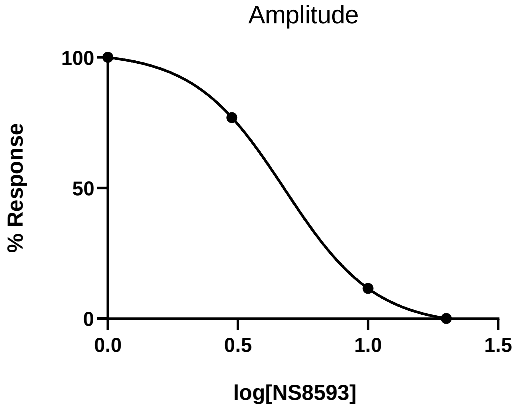
</td><td>4.78 μM</td></tr>
<tr><td>T1/2</td><td>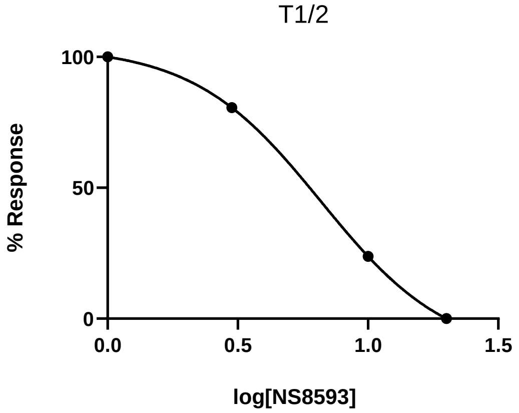
</td><td>6.60 μM</td></tr>
<tr><td>Amplitude</td><td>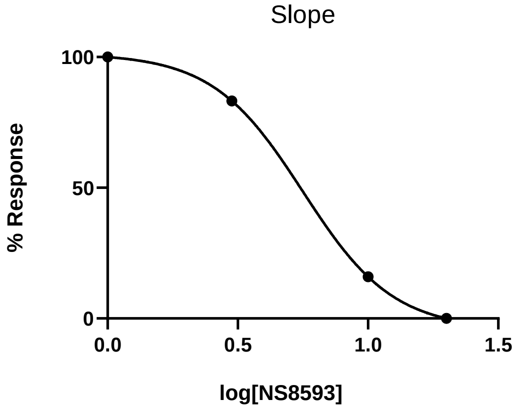
</td><td>5.62 μM</td></tr>
<tr><td colspan="3">Average = 5.67 μM, final concentration used = 6 μM</td></tr>
</table>

Figure S2. NS8593 dose responses and IC50 determination. Curves were produced using n = 4 HC not included in the main study. Abbreviations: half maximal inhibitory concentration (IC50), half-time of maximal response (T1/2).
